# A comparative study between open pre-peritoneal approach versus laparoscopic trans-abdominal pre-peritoneal approach in recurrent inguinal hernia repair: a prospective cohort study

**DOI:** 10.1007/s10029-024-02967-4

**Published:** 2024-02-01

**Authors:** P. B. A. Awad, B. H. A. Hassan, M. F. A. Kashwaa, I. M. Abdel-Maksoud

**Affiliations:** General Surgery Department, Faculty of Medicine, Ain Shams University, Cairo, Egypt

**Keywords:** Posterior approach, Laparoscopic, Hernia, Recurrent repair, Pre-peritoneal

## Abstract

**Background:**

The repair of recurrent inguinal hernias after prosthetic mesh repair is challenging due to the technical complexity and complications associated with it. As well as the increased risk of recurrence due to weakened tissues and distorted anatomy. The Posterior Pre-Peritoneal Approach yields significantly better results than the anterior approach due to its distance from previously scarred tissue.

**Objective:**

To compare the open pre-peritoneal approach and Laparoscopic trans-abdominal pre-peritoneal approach in the management of recurrent inguinal hernia which was previously managed through an open anterior approach regarding their intra-operative time, the postoperative outcomes in the form of hematoma, wound infection and finally the recurrence within 1-year follow-up.

**Patients and methods:**

The current study is a prospective cohort study, a single-center trial conducted from June 2021 to June 2022 in the general surgery department in Ain Shams University Hospitals, which included 74 patients presented with recurrent inguinal hernia who had previous open anterior approach 68(91.8%) males and 6(8.1%) females including a 1-year follow-up postoperative.

**Results:**

There were 74 patients in our study with 37 patients in each group. Group (I) underwent an open pre-peritoneal approach and group (II) underwent a Laparoscopic trans-abdominal pre-peritoneal approach. The mean age of the group (I) is 39.51 with a standard deviation of  ± 3.49. While in group (II) the mean age is 39.37 with standard deviation  ± 3.44 (*p* = 0.881). From the included 74 patients 67(91.8%) were males and 6(8.1%) were females. As regards the co-morbidities, in group (I) 17(45.9%) patients have no co-morbidities, 11(29.7%) patients have diabetes mellitus, 6(16.2%) patients have hypertension, and 3(8.1%) patients have diabetes and hypertension. Andin group (II) 26(70.3%) patients have no co-morbidities, 6(16.2%) patients have diabetes mellitus, 3(8.1%) patients have hypertension, and 2(5.4%) patients have diabetes and hypertension (*p* = 0.207). Regarding intra-operative time, the mean time in minutes in the group (I) is 63.33 with a standard deviation of  ± 11.95. While in group (II) the mean time in minutes is 81.21 with a standard deviation of  ± 18.03 (*p* = 0.015). The postoperative outcomes were assessed for 1-year follow-up in the form of hematoma, wound infection, and recurrence within 1 year. Regarding the hematoma occurred in 4(10.8%) patients in group (I). While in 2(5.4%) patients in group (II) (*p* = 0.674). The wound infection was found in 5(13.5%) patients in group(I) and zero patients in group (II) (*p* = 0.021). Finally, we followed up with the patients for about 1 year to detect the recurrence. Which was found in 3(8.1%) patients in group (I) and 1(2.7%) patient in group (II) (*p* = 0.615).

**Conclusion:**

The results of this study demonstrate that both the laparoscopic approach and the open posterior approach are effective for recurrent inguinal hernia following anterior approach mesh hernioplasty, with comparable results. Laparoscopy has been associated with a lower rate of recurrence and overall complications compared to open technique, however, it is difficult to draw definitive conclusions about the preferred option due to its lengthy learning curve and difficulty to perform. Furthermore, the results of this study confirm the previously reported positive results of the posterior pre-peritoneal for recurrent inguinal hernia, particularly when performed by experienced surgeons. Therefore, further prospective randomized population-based trials are necessary to better assess the decision-making for recurrent hernia management and the impact of specialization in abdominal wall surgery in terms of recurrence and complications.

## Introduction

The incidence of recurrent inguinal hernia is estimated to be between 0.5 and 15%, depending on many factors, such as the location of the hernia (direct or indirect), the type of hernia (mesh or non-mesh), the type of repair method (open or laparoscopic or robotic), and the type of clinical situation (elective or emergency) [1].

Furthermore, the risk of relapsing a hernia in a recurrent inguinal hernia is higher than that of a primary inguinal hernia [2], although there is limited evidence on the most effective treatment of a recurrent hernia, especially if the mesh was used in the initial procedure [3]. The most popular approach is the anterior mesh approach; however, it has the potential to re-operate through scar tissue, which carries a greater risk of complications [4]. Consequently, an open anterior approach can lead to a failure rate of up to 36%. Therefore, the posterior pre-peritoneal approach yields superior results compared to the anterior approach [5]. In a comprehensive Italian study, Campanelli et al. [6] classified recurrent hernias into three groups based on factors related to the size and location of the defect. Following this classification, he regrouped the previous surgery and selected the appropriate approach among a variety of options, including plug repair, an anterior approach, open Pre-Peritoneal (Nyhus, Stoppa, Wantz), and laparoscopic techniques. Nyhus (1960) stated that the development of the posterior pre-Peritoneal approach was first introduced to us in 1960 by Nyhus, he stated it is the preferred approach for the treatment of all recurrences of inguinal hernias [7]. However, the question should be asked whether the procedure is open or a laparoscopic approach. A recent study has indicated that the laparoscopic approach should be the preferred method of treatment for recurrent hernias, particularly in young, active, and non-obese individuals [8]. Although there is a consensus among surgeons that laparoscopic repair is the preferred option for hernias, it is not based on extensive data and the repair of hernias is typically dependent on local knowledge, cost-effectiveness, and patient preference [9]. In addition, some clinicians suggest that laparoscopic surgery is preferable in patients with a prior open repair, while those with recurrences after laparoscopic surgery should undergo open mesh repair [10]. On the other hand, laparoscopic surgery of recurrences after primary laparoscopic surgery did not provide any statistical advantage over all open techniques [9, 10].

### Aim of work

To compare the open pre-peritoneal approach and Laparoscopic trans-abdominal pre-peritoneal approach in the management of recurrent inguinal hernia repair regarding their intra-operative time, the postoperative outcomes in the form of hematoma, wound infection, and finally the recurrence within 1-year follow-up.

## Patients and methods

The current study is a prospective cohort study, a single-center trial conducted from June 2021 to June 2022 in the general surgery department in Ain Shams University Hospitals, which included 74 patients presented with recurrent inguinal hernia 68(91.8%) males and 6(8.1%) females including a 1-year follow-up postoperative. All Patients were informed both the advantages and disadvantages of each technique with the possible anesthesia type in each procedure and patients had the right to choose the technique upon their preference and if there was any contraindication for general anesthesia which was needed in the laparoscopic approach also in some cases there was unavailability to laparoscopic set.

**Inclusion criteria:** patients who are above 18 years old with recurrent inguinal hernia, accepting to do surgery and fit for surgery.

**Exclusion criteria:** patients who are less than 18 years old, refusing to do surgery, or have not done inguinal hernia repair before.

**Pre-operative:** The History of the patient including full personal history and complaint, In addition to, formal abdominal examination and examination of all hernia orifices.

*The pre-operative investigations included*:

- **Laboratory tests**: including routine complete blood count, liver profile, kidney profile, coagulation profile, blood sugar, and complete virology screen.
- **Radiological examination*:*** Pelvi-abdominal sonography, ECG, and echocardiography were performed upon request by the Anaesthesiologist when indicated.

### Patient counseling and consent

One day before the surgery the patient received a detailed explanation of the types of the surgeries and the expected postoperative complications.

The operative details were explained to help in understanding the outcome, risks, and benefits of the suggested procedure**.**

An informed consent was taken and signed by the patient and any inquiries, concerns, or doubts were discussed with the patient and a first-degree relative (upon the patient’s request). The day before surgery, all patients were instructed to have a soft diet and mineral laxatives.

#### Operative details

All procedures were performed by the same surgical team under general anesthesia or regional anesthesia. All patients had a single dose of 1 g of a third-generation cephalosporin intravenously at the induction of anesthesia.

**Fig. 1:**
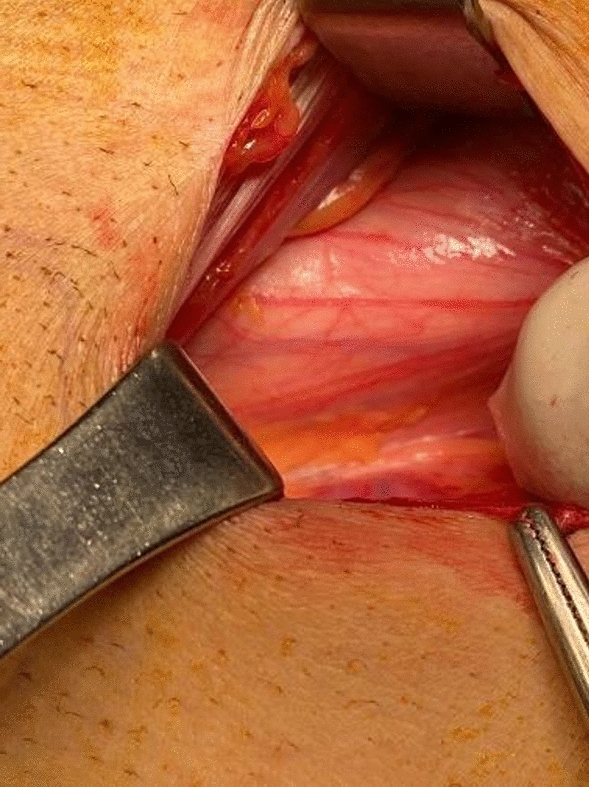
Cleaved preperitoneal space

**Fig. 2:**
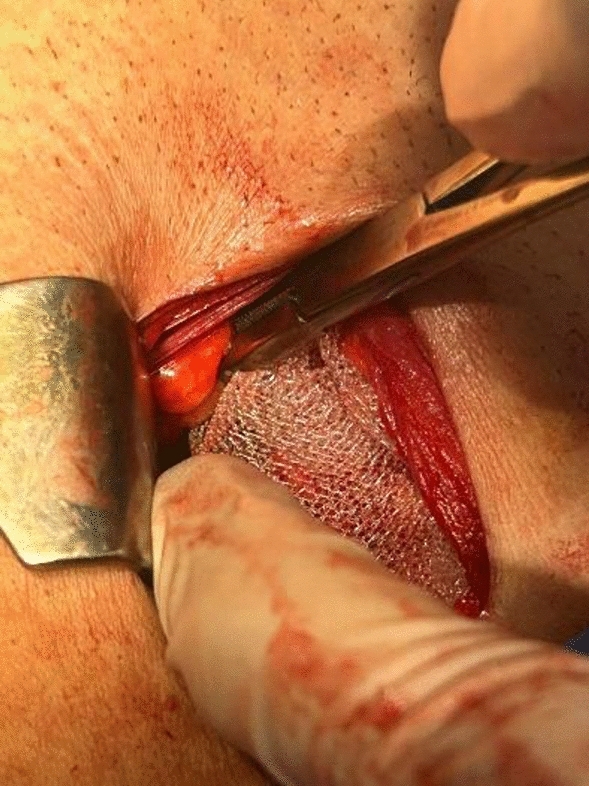
Mesh placement

*Group (I) underwent pre-peritoneal (open posterior approach)*:

- A lower abdominal transverse incision was done.
- The anterior rectus sheath was incised, and the rectus muscle reflected medially.
- The pre-peritoneal space was cleaved with blunt dissection, exposing the myopectineal orifice (Fig. 1).
- The cord was explored, and the hernias were reduced.
- A 15 ×  15 cm polypropylene mesh with a slit was inserted in the pre-peritoneal space and fixed with nonabsorbable sutures to the pubic tubercle and Cooper’s ligament.
- The mesh was passed behind the cord and manipulated to lay flat against the posterior inguinal floor overlapping the entire myopectineal orifice (Fig. 2).
- No drains were used.

**Fig. 3:**
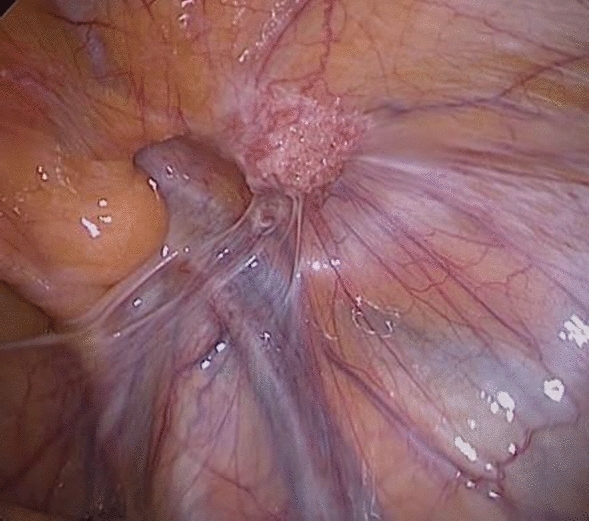
Landmarks identification

**Fig. 4:**
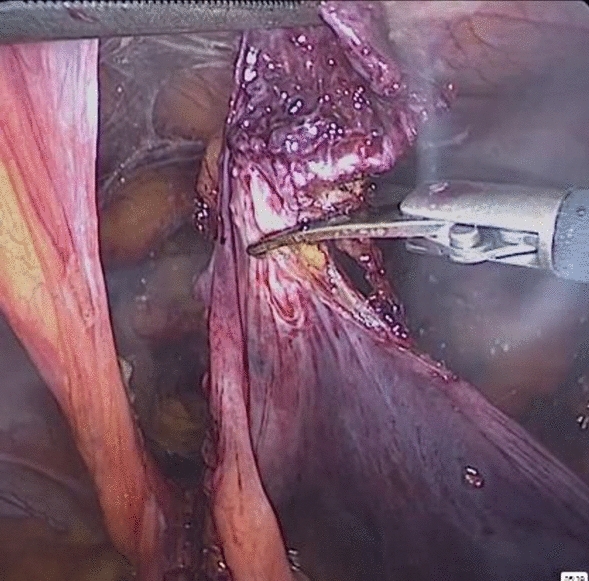
Sharp dissection to separate adhesions

**Fig. 5:**
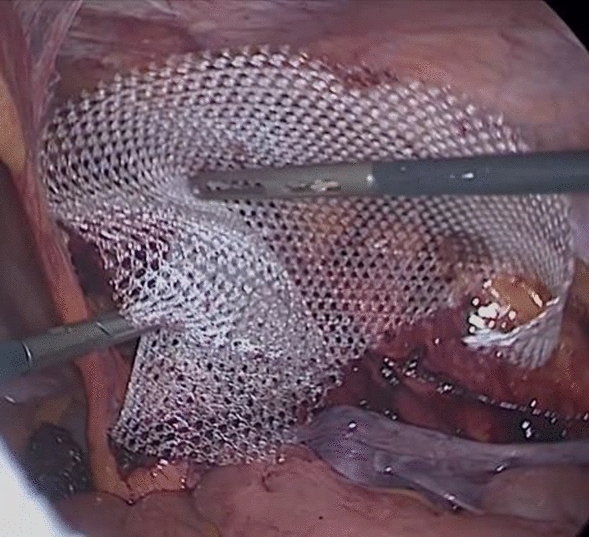
Placement of new mesh

*Group (II) underwent a Laparoscopic approach*:

- Three trocars were placed using an umbilical trocar of 10-mm and two 5-mm trocars in the left flank. Then landmarks identification was done (Fig. 3).
- The peritoneum was mobilized trans-abdominal above the hernial defect and meticulous blunt, and sharp dissection was carried out to separate the adhesions from the old mesh and the surrounding structures (Fig. 4).
- The peritoneum forming the hernia sac was pulled in, separating it from the cord structures.
- A new polypropylene mesh of approximately 15 ×  10 cm was placed over the old mesh and fixed with tacks on the pubic bone, Cooper’s ligament, and the aponeurotic arch (Fig. 5).
- The peritoneum was closed with a running suture.
- The peritoneum was deflated, and the trocar sites were closed.
- No drains were required.

Post-operative work-up and follow-up for a one-year duration. Post-operative complications in the form of hematoma, wound infection, and recurrence. Patients received oral antibiotics for one week postoperatively. Dressing of the wound was done on the second day postoperatively for all patients. All patients were trained on how to clean themselves and how to do the wound dressing. All Patients were followed up after one week, 2 weeks from discharge then every two weeks until complete healing. Then every two months complete a 1-year follow-up. None of our patients were lost during the follow-up period.

### Statistical analysis

Data were collected, coded, revised, and entered into the Statistical Package for Social Science (IBM SPSS) version 26. The data were presented as numbers and percentages for the qualitative data, mean, standard deviations, and ranges for the quantitative data with parametric distribution. **The Chi-square test** was used in the comparison between two groups with qualitative data and **the Fisher exact test** was used instead of the Chi-square test when the expected count in any cell was found less than 5. The comparison between two independent groups with quantitative data and parametric distribution was done by using **an independent t test**. The comparison between more than two groups with quantitative data and parametric distribution was done by using **One-way ANOVA**. The confidence interval was set to 95% and the margin of error accepted was set to 5%. So, the *p* value was considered significant as the following*: p* > 0.05: Non-significant (NS). *p* < 0.05: Significant (S). *p* < 0.01: Highly significant (HS).

## Results

There were 74 patients in our study 37 patients in each group. Group (I) underwent an open pre-peritoneal approach and group (II) underwent a Laparoscopic trans-abdominal pre-peritoneal approach. The mean age of the group (I) is 39.51 with a standard deviation  ± 3.49. While in group (II), the mean age is 39.37 with standard deviation  ± 3.44 (*p* = 0.881). From the included 74 patients 67(91.8%) were males and 6(8.1%) were females. As regards the co-morbidities, in group (I) 17(45.9%) patients have no co-morbidities, 11(29.7%) patients have diabetes mellitus, 6(16.2%) patients have hypertension, and 3(8.1%) patients have diabetes and hypertension. And in group (II) 26(70.3%) patients have no co-morbidities, 6(16.2%) patients have diabetes mellitus, 3(8.1%) patients have hypertension, and 2(5.4%) patients have diabetes and hypertension (*p* = 0.207) (Table 1).

**Table 1:** Shows the patients’ characteristics

Characteristic	Group I (*n* = 37)	Group II (*n* = 37)	*p*-value
Sex: Male (*n*) (%)	35 (94.6%)	33 (89.2%)	0.394
Female (*n*) (%)	2 (5.4%)	4 (10.8%)	
Mean age in years ± SD	39.51 ± 3.49	39.37 ± 3.44	0.881
Co-morbidities: (*n*)(%)			0.207
Diabetes	11 (29.7%)	6 (16.2%)	
Hypertension	6 (16.2%)	3 (8.1%)	
Diabetes and hypertension	3 (8.1%)	2 (5.4%)	
No co-morbidities	17 (45.9%)	26 (70.3%)	

Regarding intra-operative time, the mean time in minutes in the group (I) is 63.33 with a standard deviation of  ± 11.95. While in group (II) the mean time in minutes is 81.21 with a standard deviation of  ± 18.03 (*p* = 0.015). The postoperative outcomes were assessed for 1-year follow-up in the form of hematoma, wound infection, and recurrence within 1 year. Regarding the hematoma occurred in 4(10.8%) patients in group (I). While in 2(5.4%) patients in group (II) (*p* = 0.674). The wound infection was found in 5 (13.5%) patients in group (I) and zero patients in group (II) (*p* = 0.021). Finally, we followed up with the patients for about 1 year to detect the recurrence. Which was found in 3(8.1%) patients in group (I) and 1(2.7%) patient in group (II) (*p* = 0.615). Although there is no statistically significant between the two groups, there is a lower incidence in group II than in group I regarding the hematoma, wound infection, and recurrence within a 1-year follow-up (Table 2).

**Table 2:** The intra-operative time and post operative outcomes

Variables	Group I (*n* = 37)	Group II (*n* = 37)	*p*-value
Mean intra-operative time in minutes ± SD	63.33 ± 11.95	81.21 ± 18.03	0.015
Hematoma (*n*) (%)	4 (10.8%)	2 (5.4%)	0.674
Wound infection (*n*) (%)	5 (13.5%)	No cases	0.021
Recurrence within 1-year follow-up (*n*) (%)	3 (8.1%)	1 (2.7%)	0.615

## Discussion

Although the incidence of the inguinal hernia as a disease and the recurrent inguinal hernia are very high nowadays. Unfortunately, the literature does not have much data comparing pre-peritoneal approaches either open or laparoscopic. Only one trial [11] discussed that comparison since 1999 and the outcome was not as expected. Probably due to surgeons’ inexperience, or the fact that they had not finalized their learning curve.

The mean operative time was significantly longer in the TAPP group than in the OPM group the mean time was 63.33 min with a standard deviation of  ± 11.95. While, in the TAAP group the mean time was 81.21 min with a standard deviation of  ± 18.03 (*p* = 0.015). Although OPM was found to be faster this may be because it can be performed by an experienced anterior abdominal wall specialty team. However, it does not negate the fact that OPM requires a skilled hand due to the technical nature of the procedure. On the other hand, TAPP was found to be slower, which may be attributed to the setup of laparoscopy and technical burdens that needed to be adjusted related to laparoscopy. WITH REGARD TO complications, the rate of overall complications in Group I was much higher than in Group II (24.3% and 5.4% respectively), while in literature [5, 7, 11, 12] The overall postoperative complication rates in OPM were 8.2%–19.7% and may reach as high as 38% in some series [12]. Wound infection was found in 5(13.5%) patients in group (I) and zero patients in group (II) (*p* = 0.005). All cases were superficial and healed on medical treatment without the need for mesh removal. The rate of infective complications reported in the literature after open pre-peritoneal mesh repair of recurrent inguinal hernia ranges from 0 up to 10.8% [7, 11, 12]. While the rate of infective complications reported in the literature after TAAP was nearly zero. Only one study [13] mentioned early complications inform on wound infections and hematoma without discrimination was 15.3%. Regarding the hematoma occurred in 4(10.8%) patients in group (I). While in 2(5.4%) patients in group (II) (*p* = 0.001). The rate of hematoma formation reported in the literature after TAAP was from 2 up to 16% [8, 11, 14–16]. After open pre-peritoneal mesh repair ranges from 4 up to 14% [17–19]. Drainage may be recommended by some surgeons [20] ESPECIALLY IF a large, retained sac, WHERE blood CAN accumulate. In our study, drainage was not a routine, and it was only done in two of our patients. All patients who developed hematomas were treated as outpatients and resolved spontaneously without further intervention except for one patient who needed aspiration ultrasound-guided. Rather than Beets [11] study, in literature, there is consensus that when comparing recurrence rates between the two procedures, the balance is in favor of TAAP. Most of the studies [17, 18, 21] discussed the recurrence rate after OPA within the range of 0 up to 2.8%. Few papers discussed higher recurrence rates reaching up to 4.38% as Kurzer et al. [4] and Saber et al. [5] While in TAAP most of the studies [6, 14, 15, 22] reported 0% or very low incidence (1.5%) for recurrence in their studies. A recent study OF 360 patients comparing TAPP versus Lichtenstein’s repair WITH A FOLLOW-UP OF 12 years surprisingly found that the recurrence rate was high IRRESPECTIVE of surgical approach reaching 10% in both techniques [23]. In our study, we followed up with the patients for about 1 year to detect the recurrence. This was found in (3.4%) patients in the OPM group and (2.1%) patients in the TAAP group (*p* = 0.18). In conclusion, we concur with Feliu et al. [18] assertions that the rate of recurrence after the pre-operative repair is largely attributed to technical errors. As a result, it occurs at an early stage, ACCORDING TO HerniaSurge Group (2018), THAT Incorrect surgical technique is likely the most important reason for recurrence after primary IH repair [24].

## Conclusion

The results of this study demonstrate that both the laparoscopic approach and the open posterior approach are effective for recurrent inguinal hernia following anterior approach mesh hernioplasty, with comparable results. Laparoscopy May be associated with a lower rate of recurrence and overall complications compared to open technique, however, it is difficult to draw definitive conclusions about the preferred option due to its lengthy learning curve and difficulty to perform. Furthermore, the results of this study confirm the previously reported positive results of the posterior pre-peritoneal for recurrent inguinal hernia, particularly when performed by experienced surgeons. Therefore, further prospective randomized population-based trials are necessary to better assess the decision-making for recurrent hernia management and the impact of specialization in abdominal wall surgery in terms of recurrence and complications.

## Data Availability

This is a prospective cohort study including 74 patients presented with recurrent inguinal hernia 68(91.8%) males and 6(8.1%) females including a 1-year follow-up postoperative divided into two groups each group consisting of 37 patients. Group I was subjected to a pre-peritoneal (open posterior) approach and Group II was subjected to a laparoscopic approach in the management of the recurrent inguinal hernia repair. The study was done from June 2021 to June 2022 including a 1-year follow-up postoperative. The datasets used and/or analyzed during the current study are available from the corresponding author upon reasonable request.
